# Role of Heparanase and Syndecan-1 in HSV-1 Release from Infected Cells

**DOI:** 10.3390/v14102156

**Published:** 2022-09-30

**Authors:** Pankaj Sharma, Divya Kapoor, Deepak Shukla

**Affiliations:** 1 Department of Ophthalmology and Visual Sciences, University of Illinois at Chicago, Chicago, IL 60612, USA; 2 Department of Microbiology and Immunology, University of Illinois at Chicago, Chicago, IL 60612, USA

**Keywords:** HSV-1, heparanase, heparan sulfate, syndecan-1, viral release, MMPs, CREB3

## Abstract

Herpes Simplex Virus 1 (HSV-1) is a neurotropic human virus that belongs to the *Alphaherpesvirinae* subfamily of *Herpesviridae*. Establishment of its productive infection and progression of disease pathologies depend largely on successful release of virions from the virus-producing cells. HSV-1 is known to exploit many host factors for its release. Recent studies have shown that heparanase (HPSE) is one such host enzyme that is recruited for this purpose. It is an endoglycosidase that cleaves heparan sulfate (HS) from the surface of infected cells. HS is a virus attachment coreceptor that is commonly found on cell surfaces as HS proteoglycans e.g., syndecan-1 (SDC-1). The current model suggests that HSV-1 during the late stage of infection upregulates HPSE, which in turn enhances viral release by removing the virus-trapping HS moieties. In addition to its role in directly enabling viral release, HPSE accelerates the shedding of HS-containing ectodomains of SDC-1, which enhances HSV-1 release via a similar mechanism by upregulating CREB3 and COPII proteins. This review outlines the role of HPSE and SDC-1 as newly assigned host factors that facilitate HSV-1 release during a lytic infection cycle.

## 1. Introduction

Herpes simplex virus 1 (HSV-1) is a highly infectious, neurotropic human pathogen. It is a double stranded DNA virus that belongs to the *Alphaherpesvirinae* subfamily of *Herpesviridae*. It is a ubiquitous pathogen that affects 50–90% of the global population [1] and, therefore, is often regarded as an endemic that affects all age groups. Its transmission mainly occurs by oral-to-oral contact. HSV-1 infections are usually asymptomatic but can turn into ulcers or painful blisters at the site of primary infection. The infection is lifelong with symptoms reoccurring over many years. Given its ability to infect the eye, it is considered a leading cause of infection-related blindness. It causes blepharitis, conjunctivitis, retinitis, and epithelial keratitis [1]. The spread of HSV-1 to the central nervous system (CNS) when left unaddressed can also initiate fatal encephalitis in humans. HSV-1 predominantly limits itself to oral infections, but occasionally genital sores can also be caused by the virus. The frequency of genital infections caused by HSV-1 is evident from the 192 million reported cases in 2016 [2]. The primary infection by HSV-1 results in a productive lytic infection followed by the establishment of latency in the host trigeminal ganglion (TG) that has a lifelong persistence. However, there are reports suggesting other non-neuronal sites of HSV-1 latency such as corneas [3], but to date, no robust model for ocular latency has been described. For now, many in the field agree that neurons are the preferred cell type for latent infection.

The establishment of a successful infection and progression of HSV-1 pathology is largely dependent on the release of progeny virions from infected cells. The lytic cycle of HSV-1 in a host cell is represented by several key steps, namely, attachment of HSV-1 to a host cell, fusion of its envelope with the plasma membrane, entry of viral capsid into the cytosol, migration of the capsid to the nuclear membrane followed by the delivery of viral genetic material into the host nucleus, DNA replication and viral gene expression, assembly, and release of the virions from the infected cell [4]. HSV-1 entry into host cells is often regarded as an important step that governs virus induced pathology and the severity of infection. Viral entry is a rapid process. Naked capsids or intact viral particles in smooth-walled vesicles can be easily detected adjacent to the host cell plasma membrane immediately after infection [5,6]. HSV-1 has notably evolved with multiple entry mechanisms. Briefly, HSV-1 attaches to host cells by interacting with heparan sulfate (HS) chains covalently linked to the protein core of syndecans. After attachment, HSV-1 enters into the cell by an attachment/fusion process that involves viral contents including viral capsid moving into the cytoplasm through a fusion pore formation at the plasma membrane [7,8]. After the capsid has reached the cytoplasm, it is loaded onto host microtubules for transport to the nuclear membrane via cellular motors, where viral genetic material is released from the capsid into the nucleus through the nuclear pores. Subsequently, viral replication and transcription of viral genes occur in the nucleus followed by the synthesis of viral proteins in the cytoplasm. The final assembly of virions occurs in the cytoplasm, and the release of fully assembled virions marks the completion of the viral lytic lifecycle. At this stage, removal of HS is needed for the effective release and spread of virions from infected cells. Our lab has established the role of heparanase (HPSE), the only host endoglycosidase known to cleave HS moieties, in viral release via HS removal [9]. HPSE is selectively upregulated in the infected cells, and in addition to its role in enabling viral release by directly cleaving HS moieties it also accelerates the scission of HS-containing ectodomains of syndecan-1 (SDC-1), thereby further increasing HSV-1 release from infected host cells. Cleavage of SDC-1 is regulated by the increased expression of sheddase proteins known as matrix metalloproteases (MMPs) that complement increased HPSE expression in an infected state [10]. This review outlines the role of HPSE and SDC-1 as major host factors governing the release of HSV-1 during its lytic replication cycle.

## 2. HSV-1: An Overview

### 2.1. Structural Organization of HSV-1

**Figure 1:**
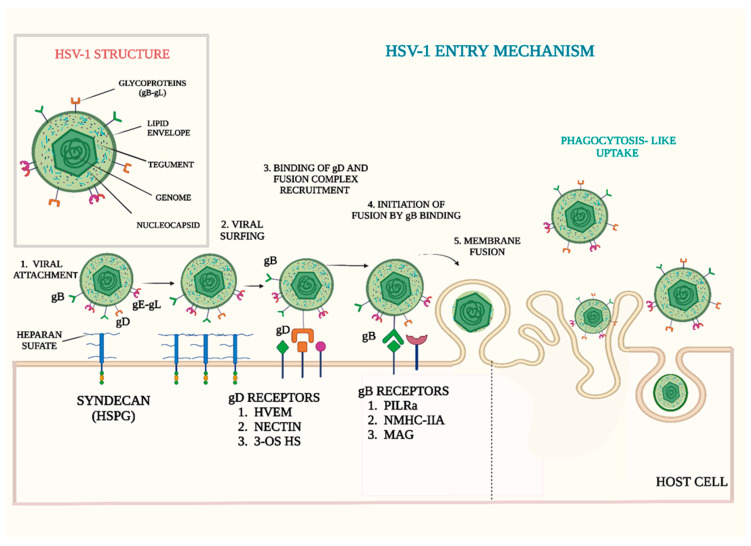
HSV-1 ENTRY MECHANISMS. Top right: HSV-1 structure constitutes outer lipid envelope that contains 12 glycosylated (gB, gC, gD, gE, gG, gH, gI, gJ, gK, gL, gM, gN) and three unglycosylated (UL20, UL45, US9) proteins, inner tegument proteins and nucleocapsid enclosing genome. Bottom center: HSV-1 entry requires receptor-binding glycoprotein gD, the heterodimer gH/gL, and the fusogen gB. Different glycoproteins utilize different host receptors such as gD receptors (HVEM, herpes virus entry mediator, Nectin-1), 3-OS-HS, 3-O-sulfated-heparan sulfate, gB receptors (PILRα, paired immunoglobulin-like type 2 receptor alpha, NMHC-IIA, non-muscle myosin II, MAG, myelin associated glycoprotein) for entry and cell to cell spread. gC makes the first contact with the host cell, binding to heparan sulfate (HS) proteoglycans on the cell surface. In some cases, HSV-1 travels down filopodia-like membrane protrusions to reach the cell body for internalization. This extracellular transport of virions is called “surfing”. The glycoprotein gB regulate viral surfing, as it binds to the HS attached to the syndecan (HSPG). Once the virus is bound, it travels unilaterally to the cell surface, where gD proceeds to bind with one of its four receptors (discussed above) and the process of virus penetration and membrane fusion ensues, or the virus can enter the cell by phagocytosis-like uptake [Created in bioRender].

The family of herpesviruses has more than 100 members with their hosts ranging from oysters to human beings. HSV-1 is a member of the alphaherpesvirus subfamily, which is comprised of neurotropic viruses known to cause multiple diseases such as mucocutaneous lesions, ocular keratitis, skin infections as well as meningitis and encephalitis [11]. These enveloped viruses share a common virion structure and analogous proliferation approaches [12]. Their double-stranded DNA genome, which is around 150 kbp in size, is packed into an icosahedral shaped capsid. A layer of tegument proteins surrounds the capsid. The capsid and the tegument are confined within a lipid-bilayer envelope as shown in Figure 1 [13]. The icosahedral capsid is around 125 nm in diameter and encloses a single copy of the genome that encodes almost 80 open reading frames. The virion envelope is decorated with viral glycoproteins that are known to facilitate its entry into host cells [14]. The genome is organized into covalently linked sections termed Long (L) or Short (S) segments based on their comparative lengths. Each of these segments encompasses unique sequence regions (UL or US) that are bordered by inverted repeat sequences. The genes present in the unique regions exist as a single copy in the genome, but genes encoded in the repeat regions, such as ICP0 or ICP4, occur as two copies [15]. Around 50% of the genes encoded by the HSV-1 genome are considered to be non-essential in viral replication in cell-culture [16,17]. The genes of HSV-1 responsible for DNA replication, gene and capsid transcription, capsid protein translation, packaging DNA proteins and some envelope glycoproteins are regarded as essential genes. Conversely, non-essential genes regulate nucleic acid metabolism, help in evading host immune responses, assist in optimal replication of the virus and primary envelopment, and viral pathogenesis [17].

### 2.2. Entry of HSV-1 into Host Cells

In their quest to complete their lytic cycle, herpesviruses attempt to completely control host cell machinery causing cell lysis at the end of the infection. HSV-1, being an enveloped virus, penetrates into the cell by inducing fusion of the viral envelope with the host cell membrane [13,18,19]. Depending upon the cell type, HSV-1 may also enter through a phagocytosis-like endocytic pathway in a pH-dependent or pH-independent manner [20,21].The virions are internalized and transported through the early endosomes in host cells. The slightly acidic pH of the endosome stimulates structural modifications in the viral fusion proteins that allow for the fusion of the viral envelope and vesicular membrane, causing the nucleocapsid to be released from the vesicle into the cytoplasm [20,21]. The fusion of the plasma membrane and viral envelope is triggered by a cascade of receptor-ligand interactions that ultimately induces fusion and dispersion of the nucleocapsid into cytoplasm. This pathway is commonly used by HSV-1 to enter into Vero cells and is facilitated by different viral glycoproteins such as gB, gC, gD, gH and gL. Initially, binding of HSV-1 is mediated by the association of gB and/or gC to HSPGs present on the host cell surface [7]. After this initial binding or attachment, gD interacts with its host receptor leading to conformational changes in its structure that are thought to act as a signal for gH/gL activation. This is followed by the formation of a gH-gL/gD-receptor complex that triggers a structural change in gB that promotes viral membrane insertion into the host cell membrane. The HSV-1 gD receptors [22] HVEM (a member of tumor necrosis factor receptor family), and Nectin-1 (belonging to immunoglobulin superfamily of host receptors) are shown in Figure 1. Interestingly, in the absence of HVEM and nectin-1, specific modifications in HS, termed 3-O-sulfated HS, can act as potent receptors for HSV-1 gD [23].

### 2.3. Replication and Translation of HSV-1

After entry, HSV-1 releases its genetic material into the cytoplasm of the cell that ultimately reaches the nucleus for replication. Successful entry of the virus into the host cell also marks the halt of normal cellular functions and host immune responses to support the initiation of the viral replication process. To reach the nucleus, the capsid is driven by cellular motors on microtubules and delivered to the nuclear pores for uncoating of the viral genome [20,24] After viral genome entry, a cascade of viral gene expression begins. HSV-1 genes are expressed in three chronological phases: immediate early (α), early (ß) and late (ɣ). HSV-1 exploits host RNA polymerase II for synthesizing all its viral transcripts. Host proteins are vital for the formation of viral mRNAs, whereas viral proteins are indispensable for the induction and augmentation of transcription of particular genes. The synthesis of an entire range of viral gene products that are required for a productive infection necessitates the coordinated action of several proteins [15,25]. The genes that are transcribed immediately after infection are immediate early genes. The tegument protein VP16 is a crucial primary regulator of immediate early gene transcription which includes the genes encoding ICP0, ICP4, ICP22, ICP27, and ICP47. These are known to activate the transcription of early genes that participate in viral genome replication. Replication of viral DNA promotes the expression of late genes that are known to encode structural proteins. These proteins also include HSV-1 glycoproteins that participate in subsequent infections.

## 3. Assembly and Release of HSV-1 from Infected Cells

Following viral gene expression and genome replication, the packaging of HSV-1 genetic material into preformed capsids in the nucleus ensues [26]. The packaging in capsids happens via a pUL6 portal and necessitates the activity of the terminase complex [27]. The translocation of viral genome in the procapsid is an ATP-dependent process. The packaging of viral DNA is accomplished by the interactions of a terminase complex with pac sequences present in the terminal repeat region at the open end of viral DNA [28,29]. Upon successful completion of DNA packaging, the terminase complex cuts the concatemeric viral DNA to pack a single genome copy in each progeny virion. The procapsid also undergoes proteolytic processing that leads to prominent conformational changes resulting in the reorganization of the procapsid (spherical) into the stable (icosahedral) capsid [30].

**Figure 2:**
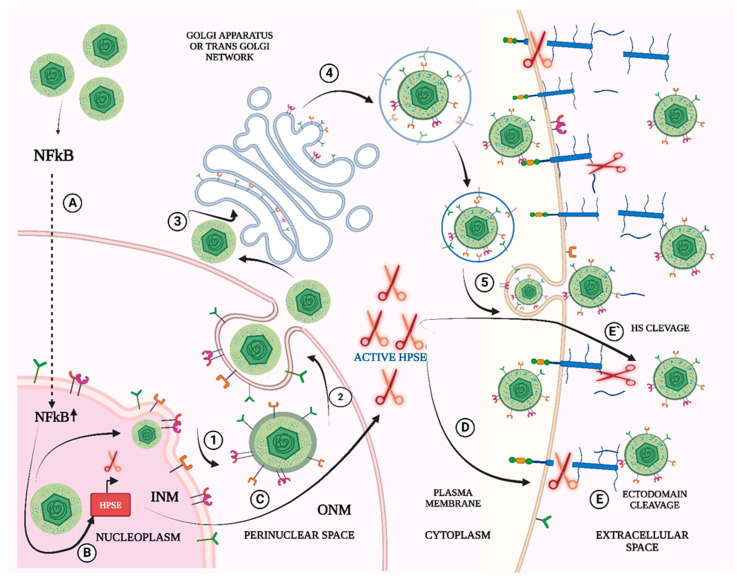
OVERVIEW OF HSV-1 RELEASE. (**1**–**2**) After capsids are formed in the nucleus, they bud into the inner nuclear membrane (INM) (primary envelopment) to form an enveloped particle in the perinuclear space. These particles fuse with the outer nuclear membrane (ONM) (de-envelopment) and are released into the cytoplasm, leaving the envelope in the ONM. (**3**–**4**) In the cytosol, capsids bind onto trans Golgi network and bud into cytoplasmic membranes (secondary envelopment) (**5**) and enveloped virions are ready to be secreted from cells (release). HPSE IN VIRAL RELEASE. (**A**) Entry of HSV-1 into the host cells stimulate the translocation of NF-kB from the cytoplasm to the nucleus. (**B**–**D**) NF-kB translocation further upregulate heparanase (HPSE) in the cytoplasm that travels to the extracellular membrane and aids in (**E**) heparan sulfate cleavage and (**E’**) Cleavage of syndecan-1 ectodomain that aids in enhanced release of virions from the infected host cell. [Created in bioRender].

On completion of genome packaging and capsid assembly inside the nucleus, the subsequent nucleocapsids must leave the boundaries of the nuclear envelope but face the obstacle of being surrounded by a phospholipid bilayer. The mechanism for crossing the bilayer is a multistep process. The first step of nuclear exit is envelopment at the inner nuclear membrane and then fusion with the outer nuclear membrane [30,31].The final assembly of the virions occurs in the cytoplasm. The trans Golgi network plays a crucial role in final assembly of the virions (Figure 2). The ultimate stage in herpesvirus virion assembly requires a membrane cleavage for the separation of progeny virions from the neighboring host-cell membrane. Herpesviruses are known to exploit the membrane cleavage activity of the cellular endosomal sorting complex (ESCRT) routinely. This process also leads to concurrent synthesis of the viral envelope and packing into vesicles that finally fuse at the plasma membrane to release enveloped virions [32].

The release of HSV-1 from infected cells is known to be affected by different host and viral factors with HSV-1 exploiting different host factors to facilitate its release. Among the major factors known to hinder the release of progeny virions are the same HSPGs that initially facilitated viral entry. The HS moieties need to be cleaved off for viral release. HSV-1 exploits the host machinery by selectively upregulating HPSE, a HS-degrading enzyme of the host to facilitate its release. Recent findings have demonstrated that HPSE acts as a molecular switch between a virus-permissive ‘attachment mode’ of host cells to a virus-deterring ‘detachment mode’ [9]. In addition to its ability of directly facilitating viral release by HS removal, it also enables the cleavage of HS side chains containing SDC-1, a transmembrane proteoglycan, thus accelerating HSV-1 release from infected cells. 

## 4. HPSE: A Key Enzyme Governing HSV-1 Release and Associated Pathology

HPSE is an endo-β-D-endoglycosidase that has a crucial role in the degradation and remodeling of the extracellular matrix (ECM). It is the only mammalian enzyme that has the potential to cleave HS moieties [19,33]. The cellular function of this enzyme corresponds to neovascularization, cell proliferation, inflammation, angiogenesis and autoimmunity, including the relocation of vascular endothelial cells and activated immune cells [34]. HPSE is highly conserved and shares similar sequences across diverse species such as human, rat, mouse, cow, chicken, mollusks and zebra fish. It is a multifunctional protein bestowed with enzymatic as well as non-enzymatic functions that participates in major human pathological processes [35]. The non-enzymatic roles of HPSE are largely represented by the stimulation of PI3K/Akt axis and signaling, DNA damage response, endothelial cell migration (p38-dependent), and augmentation of VEGF [19,33]. Initially, it is synthesized as a 65 KDa latent precursor that undergoes proteolytic processing to yield 8 KDa and 50 KDa protein subunits that heterodimerize to produce the active enzyme. The active form of HPSE has been found to be associated with a variety of diseases, most notably cancer [24,36]. Several recent studies support a role of active HPSE in modulating the life cycles of numerous human viruses including HSV-1, respiratory syncytial virus (RSV), human papilloma virus (HPV), the porcine respiratory and reproductive syncytial virus (PRRSV) and dengue virus [37,38]. HPSE’s role in viral release is analogous to influenza virus neuraminidase, which cleaves off sialic acid residues to facilitate viral release [39].

### 4.1. HSV-1 Infection Upregulates HPSE

HPSE is selectively upregulated in HSV-1 infected cells. This was established when attempts to develop novel interventions against HSV-1 using anti-HS peptides [40]. resulted in an important and unexpected observation that there was a sequential decline in the HS levels on the surface of HSV-1 infected HCE cells [9]. Interestingly, during the primary stages of infection, an increase in the levels of HS facilitates viral attachment to host cells [41]. However, the continued progression of viral infection marks a significant decline in the levels of HS to the extent that HS seems to disappear during the last phase of infection. Additionally, the degradation of HS increased with a respective increase in the multiplicity of infection, indicating its direct correlation to the levels of infection [9]. To identify the possible cause of HS degradation, the study found a significant increase of HPSE transcripts as well as the protein levels of this enzyme. Recently, Hopkins et al. [42] also reported the transcriptional upregulation of HPSE after HSV-2 infection in a vaginal epithelial cell model that shows increased HPSE activity during the end phase of the infection. Both studies show that overexpression of HPSE leads to significantly enhanced viral release. In contrast, the knockdown of HPSE causes a decline in the viral titer from culture supernatant [9]. The authors also established that only functional HPSE is essential for efficient viral release, as transfection with functionally impaired HPSE mutants impeded viral release. Overexpression of a constitutively active form of HPSE, known as GS3-HPSE, in murine corneas worsened the herpetic disease in vivo. The GS3-HPSE-transfected mice exhibited enhanced ocular discharge, erythema and periorbital edema, all indicating signs of severe herpetic pathology [18]. To further support an important role of HPSE in viral release, the use of OGT 2115, a pharmacological inhibitor of HPSE, blocks viral release and, therefore, demonstrates a good translational potential as an antiviral agent [18]. Interestingly, another host enzyme, Cathepsin L, which is required for the conversion of pro-HPSE to its active form, was later shown to have a role in viral release [42].

### 4.2. NF-κB Drives Increase in HPSE Levels

Increased expression of HPSE coupled with a decline in the surface levels of HS indicates that a viral or host factor is likely responsible. One such mechanism is proposed by Hadigal et al. [9] which states that during HSV-1 infection, the host factor NF-κB is translocated into the nucleus where it binds to the HPSE promoter and enhances the transcription of HPSE in HCE cells. The authors observed a progressive and significant increase in the transcripts of NF-κB with infection. There was also enhanced nuclear translocation of the p65 subunit at 24 and 36 hpi. The authors confirmed the nuclear translocation of NF-κB using immunofluorescence microscopy where translocation was clear in response to GFP-tagged viral infection. Furthermore, Western blot analysis of nuclear and cytoplasmic fractions indicated a decline in NF-κB expression in the cytoplasmic fraction in response to viral infection, whereas the expression of NF-κB in infected cells was significantly increased in the nuclear fraction [18]. The treatment of uninfected HCE cells with Betulinic acid, an inducer of NF-kB, also promoted its nuclear translocation followed by upregulation of HPSE mRNAs. This clearly indicated that NF-kB activation during HSV-1 infection plays a role in HPSE induction. To add more evidence to the data, the authors [9,42] overexpressed a dominant-negative mutant of NF-kB, IkBa (S32A/S36A), which is incapable of being phosphorylated and degraded. As expected, the expression of dominant negative mutant inhibited the activation of NF-kB and its translocation to the nucleus. Subsequently, the authors reported a decline in HPSE expression by qPCR and luciferase reporter assays. In parallel, to assess any possible causality of viral transcription factors in HPSE upregulation, the authors transfected HCEs with expression plasmids for the transcriptional regulators: ICP0, ICP4, ICP22, and VP16 and observed no significant differences in HPSE expression.

### 4.3. HPSE Induces Activation of β-Catenin

HPSE mediates multiple non-enzymatic activities by stimulating intracellular signaling to promote phosphorylation of various proteins such as Akt, ERK, p38, and Src [43,44]. Recently, our group established a unique function of HPSE as a crucial regulator of β-catenin. The latter is important for regulation and coordination of cell-to-cell adhesion and gene transcription. The authors of this study, ref. [45] reported that HSV-1 infection in HCE cells triggers the nuclear translocation of β-catenin. The cell culture results were further verified in a murine model. In HSV-1-infected mice, extensive upregulation of β-catenin was detected in conjunction with its nuclear translocation in the corneal epithelium. The nuclear localization of β-Catenin was found to be associated with enhanced viral protein production [45]. HPSE was found to play a vital role in nuclear translocation of β-Catenin as HCE cells expressing constitutively active HPSE (GS3-HPSE) displayed increased nuclear localization of β-catenin whereas the cells lacking HPSE were found to exhibit lower β-catenin levels during HSV-1 infection. HSV-1 induced activation of AKT leads to the stabilization of β-catenin, which plays a pro-viral role. In addition, β-catenin was also found to promote viral replication as pharmacological inhibition of β-catenin displayed a significant suppression of viral replication. The gene silencing of β-catenin using siRNA also hindered viral protein production. Therefore, HSV-1 appears to utilize HPSE induced activation of β-catenin that supports viral replication.

### 4.4. HSV-1 ICP34.5 As a Possible Viral Factor for The Upregulation of Cellular HPSE 

The identification of viral factors implicated in infection induced HPSE upregulation is important. HSV-1-infected cell protein (ICP) 34.5 has been involved in governing interferon responses and viral release [46,47] HSV-1 lacking γ 34.5 gene (mutant virus) displayed a compromised ability of upregulating HPSE mRNA in HCE cells [18]. The authors also confirmed a loss of HPSE promoter activity in mutant virus infected HCE cells. To provide further connection the co-transfection of HPSE-Luc with γ 34.5 gene increased the expression of luciferase driven by the HPSE promoter. In addition to cell culture results, similar observations were also made in porcine corneas [18]. Not only was HPSE is upregulated by the virus expressing the wild-type copy of γ 34.5 gene, but it was also found that HPSE and HSV-1 co-localize in porcine corneas. This connection is also supported by the fact that γ 34.5 is expressed during later stages of infection and HPSE upregulation coincides with its expression.

### 4.5. Involvement of CREB3 in Enhancing HPSE Induced Viral Release

HSV-1 is known to modulate numerous host factors to improve its replication and release from the host cells. Recently, Yadavalli et al. [48] reported that cyclic-AMP-responsive element-binding protein 3 (CREB3) acts as an important mediator in HPSE-assisted release of HSV-1 from infected cells. A transient upregulation in the levels of CREB3 promoted a corresponding increase in the extracellular viral release from the infected HCE cells. To unfold the molecular mechanisms, the authors transfected the cells with HPSE and CREB3 before infecting them with HSV-1. Surprisingly, the authors observed that overexpression of HPSE stimulated CREB3 levels, whereas overexpression of CREB3 did not stimulate HPSE expression. To check whether CREB3 expression also decreases with HPSE, the authors utilized HPSE knockout mouse embryonic fibroblast (MEF) cells. Western blotting analysis revealed that infection with HSV-1 triggered an increase in the expression of CREB3 after infection in both the cell types. However, the expression of CREB3 was observed to be much lower in HPSE knockout cells. Therefore, CREB3 was upregulated with the overexpression of HPSE but not vice versa. It is known that coat protein complex II (COPII) facilitates HPSE trafficking and CREB3 regulates COPII vesicle formation [49]; therefore, the authors evaluated COPII transcripts during upregulation of CREB3. Interestingly, the upregulation of CREB3 was found to be positively correlated with the upregulation of COPII-associated transcripts:SEC13, SEC23A, SEC24B, SEC24D, and SAR1B, and to further confirm the results, the authors co-transfected HCE cells with CREB3 and HPSE, and observed maximum viral release as equated to either treatment alone, establishing CREB3 as a key player in HPSE-facilitated HSV-1 release [48].

## 5. Role of SDC-1 Shedding in HSV-1 Pathogenesis

Syndecans are a small family of transmembrane proteoglycans that network with a variety of ligands through their core proteins and glycosaminoglycan (GAG) chains, which includes HS [50]. They are considered endocytic receptors that are internalized via clathrin-dependent processes [51]. Many lipoproteins are known to bind to SDC-1 on hepatocytes and then internalized in a flotillin-dependent manner [52]. There are four types of syndecans, namely syndecan-1 to -4, based on their discovery. Structurally, the syndecans are composed of an N-terminal ectodomain containing numerous consensus sequences for GAG association, a transmembrane domain with a short protease cleavage site proximal to the ectodomain and a cytoplasmic domain (short) that is the C-terminal. The cytoplasmic domain has C1 and C2 regions that are highly conserved and are divided by a variable (V) region. The intermediate V region between C1 and C2 regions varies among every syndecan member still conserved across species, thus facilitating the syndecan-specific intracellular functions [50]. These proteins are quite divergent in structure but have properties in common. All the transmembrane proteoglycans have roles as coreceptors, and they commonly associate with growth factor receptors or adhesion receptors. Syndecans are expressed on different cell types and locations at different times and levels and, hence, likely perform specific functions in vivo [53,54]. For example, SDC-1 is detected at the four-cell stage during mouse development, indicating that expression is zygotically activated. In adult tissues, SDC-1 is predominantly expressed ubiquitously on the surface of epithelial cells as the major HS carrying HSPG. It is composed of large HS chains covalently attached to its extracellular domain and a short cytoplasmic tail that interacts with a number of signaling enzymes as well as adaptors. It is a known mediator of micropinocytosis that promotes tumor cell growth [55] While it has crucial regulatory roles in multiple genetic processes including inflammation and angiogenesis, its direct role in viral pathogenesis and more specifically, viral release, is not as well understood [53,54,56,57,58,59]. Likewise, the phenomenon of SDC-1 ectodomain shedding and a role for HPSE in this process is well studied in malignancies, but its critical role during HSV-1 infection is still emerging.

### 5.1. SDC-1 Shedding Is Upregulated upon Infection

HSV-1 entry is facilitated via interaction of its glycoproteins, specifically gB and gC, with HS moieties present on HSPGs. Cell surface syndecan expression increases during viral entry [41]. However, during the later stages of infection the cell surface expression of syndecans decreases and this phenomenon is connected to viral release [10,60]. In order to understand the dynamics of HSV-1 infection and SDC-1 expression, Hadigal et al., 2020, utilized HCE cells that are clinical targets for HSV-1 infection. The authors observed significant decrease in cell surface SDC-1 expression with infection. Similar losses in SDC-1 levels were also seen in human cornea organ cultures after HSV-1 infection. In both cases, higher levels of SDC-1 ectodomains were found in culture supernatants, suggesting enhanced shedding. These data led to the conclusion that SDC-1 shedding is enhanced with HSV-1 infection.

There exists a possibility that enhanced SDC-1 shedding is due to an increase in its synthesis in the infected cells, as shown in HeLa cells by Bacsa et al. [41], but this was ruled out in corneal cells during HSV-1 infection in vitro, as has been demonstrated by Hadigal et al. [10]. They analyzed both transcripts and protein levels in HSV-1 infected HCE cells. They observed no substantial increase in SDC-1 transcripts or protein synthesis during infection. They concluded that the enhanced syndecan shedding observed during infection was not a result of its transcriptional upregulation. Further, the differences in the HCE cells and HeLa cells depicts its cell specific nature.

### 5.2. Active HPSE Drives SDC-1 Shedding

Recently, multiple reports have established the relation between HPSE and SDC-1 ([10,60] In addition to its role in ECM remodeling via releasing HS chains, HPSE also enhances virulence by promoting ectodomain shedding of SDC-1. This novel function of HPSE further underscores the significance of HSPGs and HPSE in driving HSV-1 release, virulence, cell to cell spread and thus pathogenesis.

Multiple reports suggest different mechanisms of SDC-1 shedding. HPSE is unique in its interaction with SDC-1 shedding due to its extracellular mode of action. As HPSE is converted into its active form and upregulated during corneal HSV-1 infection, it has been proposed to have direct/indirect effect on SDC-1 shedding [10]. Our lab recently showed that HPSE-transfected cells had less SDC-1 expression (on average 50% less) on their cell surfaces compared to the cells transfected with an empty vector [10].

### 5.3. Upregulation of SDC-1 Cleaving Sheddases during HSV-1 Infection

Syndecan shedding is a result of the cleavage that occurs through the direct activity of extracellular proteases, i.e., sheddases. The mechanism that targets upregulation and activity of sheddases, though studied in various fields, remains unknown.

Matrix metalloproteinases (MMPs) are a family of endopeptidases with 23 members that are sheddases capable of cleaving syndecans from the cellular surface [61]. Past studies have demonstrated that different MMPs stimulate upregulation of HPSE. Interestingly, lowering the MMP-9 levels has shown to reduce herpes simplex keratitis in both lab conditions as well as animal models [62,63]. Hadigal et al. [10] quantified the levels of MMP transcripts in HPSE overexpressed HCE cells versus control cells. Among five different MMPs tested, the transcripts of MMP-3 and MMP-7 displayed significant increase in HPSE overexpressed cells. Since HSV-1 is known to upregulate HPSE expression and HPSE upregulation in non-infected cells and further stimulate MMPs, the authors tested whether HSV-1 infection could stimulate the expression of these MMPs. Upon infection with HSV-1, they observed a similar increase in MMP-3 and MMP-7 transcripts in HCE cells. They also found evidence that MMP-3 and MMP-7 transcripts upon HSV-1 infection corroborate with their enhanced expression on the cell surface These results clearly indicate a cascade of events stimulated by HSV-1 infection leading to HPSE upregulation that further led to increase in MMP-3 and MMP-7 expression, while suggesting their migration to the cell surface to assist SDC-1 shedding [61,64].

Although it has been specifically shown with the HCE cell line that MMP-1 and MMP-3 transcripts increase during viral entry, the increase is likely time dependent. There are multiple reports suggesting that MMP transcripts decrease with HSV-1 infection during early infection time points i.e., up to 8 hours post infection, and the virion host shutoff (*vhs*) RNase plays a key role in this process. The vhs protein is responsible for efficiently cleaving host mRNAs [65,66].We believe that the sheddases transcripts initially decrease because of high vhs activity but eventually their expression is enhanced due to strong transcriptional activity favoring proviral proteins.

### 5.4. Enhanced Viral Release during Agonist Induced SDC-1 Shedding

Thrombin, PMA, and epidermal growth factor (EGF) are some of the reported compounds involved in enhanced SDC-1 shedding [61,64]. EGF and thrombin are compounds involved in wound healing that accelerate syndecan shedding through receptor tyrosine kinases (RTK) and by activation of G-protein coupled receptors (GPCR). Recent studies have demonstrated that thrombin, PMA and EGF mediated shedding of SDC-1 can enhance HSV-1 release from infected cells [60]. Collectively, they showed that along with HS cleavage, the whole HSPG specific SDC-1 shedding is upregulated upon HSV-1 infection in both HCE and HeLa cells. The authors further proposed that this enhanced SDC-1 shedding might be mediated by HPSE stimulation.

Specifically, the authors treated HCE and HeLa cells with PMA (phorbol 12-myristate 13-acetate), a known shedding agonist of SDC-1, thrombin (protease), and a growth factor (EGF). They found that the agonists accelerated SDC-1 shedding in both HeLa and HCE cells. They also demonstrated that increased SDC-1 expression in the supernatant was due to its loss from the cell surface. Their plaque assay results showed that PMA, thrombin, and EGF treated cells resulted in an enhanced HSV-1 release and increased number of extracellular viruses. Thus, they were able to demonstrate that agonist induced SDC-1 shedding leads to more viral release.

## 6. Conclusions

Recent studies in the herpesvirus pathogenesis field have identified important roles for HPSE and SDC-1 in HSV-1 release. The data emphasize that HPSE is a key host factor that directly removes HS during viral release, and indirectly, regulates b-catenin and CREB3 activities as well as SDC-1 ectodomain shedding to promote viral release. In parallel, SDC-1 ectodomain can also be shed in response to other stimuli, promoting viral exit. The HPSE/syndecan axis has the potential to delineate host mechanisms that contribute directly to the release of several different pathogenic human viruses that use HS for attachment and entry into their host cells. While the host factors promoting HSV-1 egress are being increasingly well studied, imperative challenges persist, including characterizing any cell type-specific differences in virus release, the impact of HPSE activity and syndecan shedding in preventing viral entry into nearby cells, and the direct significance of HPSE and syndecan in cell-to-cell spread of HSV-1 in animal models. The aftermath of the highly pathogenic environment created by enhanced HPSE release and syndecan shedding is also poorly understood. In addition, the other members of HPSE and syndecan axis, for instance MMPs, also require additional studies for their roles in HSV-1 release. These and other developments in viral release mechanisms will help identify the best strategies for curbing HSV-1 release for therapeutic benefits and delineating the pathogenic roles of HPSE and syndecan during HSV-1 infection.
